# Rapid Decline of Follicular Lymphoma-Associated Chylothorax after Low Dose Radiotherapy to Retroperitoneal Lymphoma Localization

**DOI:** 10.1155/2014/684689

**Published:** 2014-05-07

**Authors:** Lien Van De Voorde, Ben Vanneste, Jacques Borger, Esther G. C. Troost, Philo Werner

**Affiliations:** ^1^Department of Radiation Oncology (Maastro Clinic), School for Oncology (GROW), Maastricht University Medical Centre, 6229 ET Maastricht, The Netherlands; ^2^Department of Oncology, 5912 BL Viecuri, The Netherlands

## Abstract

Chylothorax is caused by disruption or obstruction of the thoracic duct or its tributaries that results in the leakage of chyle into the pleural space. A number of interventions have been used to treat chylothorax including the treatment of the underlying disease. Lymphoma is found in 70% of cases with nontraumatic malignant aetiology. Although patients usually have advanced lymphoma, supradiaphragmatic disease is not always present. We discuss the case of a 63-year-old woman presenting with progressive respiratory symptoms due to chylothorax. She was diagnosed with a stage IIE retroperitoneal grade 1 follicular lymphoma extending from the coeliac trunk towards the pelvic inlet. Despite thoracocentesis and medium-chain triglycerides (MCT), diet chylothorax reoccurred. After low dose radiotherapy (2 × 2 Gy) to the abdominal lymphoma there was a marked decrease in lymphadenopathy at the coeliac trunk and a complete regression of the pleural fluid. In this case, radiotherapy was shown to be an effective nontoxic treatment option for lymphoma-associated chylothorax with long-term remission of pleural effusion.

## 1. Introduction

Chylothorax is a rare cause of a large pleural effusion and progressive respiratory failure. In about 18% of the cases of chylothorax, advanced Hodgkin's or non-Hodgkin's lymphoma is the underlying cause and thus accounts for 70% of cases with nontraumatic malignant aetiology [1–10]. Nowadays the diagnosis of lymphoma is made in an earlier phase with often prompt initiation of effective therapy. In the literature, we only found a limited number of case reports regarding this presentation [11]. Unfortunately, the rarity with which lymphoma causes chylothorax and the lack of an adequate animal model make investigations regarding the pathophysiology difficult. There are two assumed mechanisms by which chyle may influx into the pleural cavity. First, increased pressure in the thoracic duct may cause retrograde flow of chyle via the lymphatics of the parietal pleura into the pleural cavity. Another mechanism is thoracic duct rupture due to infiltration of the duct by a lymphoma which causes the susceptibility of the duct to rupture [12]. Chyle can accumulate in either the left or right pleural space, depending on where the thoracic duct is disrupted. Around the fifth thoracic vertebra, the thoracic duct crosses to the left side of the vertebral column, where it continues ascending behind the aortic arch into the neck above the clavicle, and terminates into the venous circulation, where the left subclavian and jugular veins combine to form the brachiocephalic vein [13]. Patients with chylothorax rapidly suffer from malnutrition and immunodeficiency because of the loss of fat and lymphocytes [1, 12]. Chylothorax often becomes a chronic problem and its course does not always reflect the successful treatment of lymphoma with chemotherapy [1, 2, 14–16]. Furthermore, in most of these previously reported cases mediastinal lymphadenopathy was present or the radiation field included the mediastinum [1]. We discuss the case report of a patient diagnosed with stage IIE follicular lymphoma located at the celiac trunk presenting initially with a chylothorax. Follicular lymphoma, a B-cell lymphoma, is the most common indolent (slow-growing) form of non-Hodgkin lymphoma, accounting for approximately 20 percent to 30 percent of all non-Hodgkin lymphomas [17]. Because of its relapsing and remitting course, it is usually not possible to achieve definitive cure [18]. The two best measures of outcome are the follicular lymphoma international prognostic index (FLIPI) and tumor grade [19]. From “watchful waiting” to high dose systemic therapy, numerous treatment options have been proposed for patients with follicular lymphoma. Given the potential long-term survival of these patients, treatment with favourable side-effect profile and minimal long-term risks is desired [20]. To our knowledge, this is the first case report in which low dose radiotherapy was planned as primary treatment to obtain a complete remission of the pleural effusion.

## 2. Case Presentation

**Figure 1:**
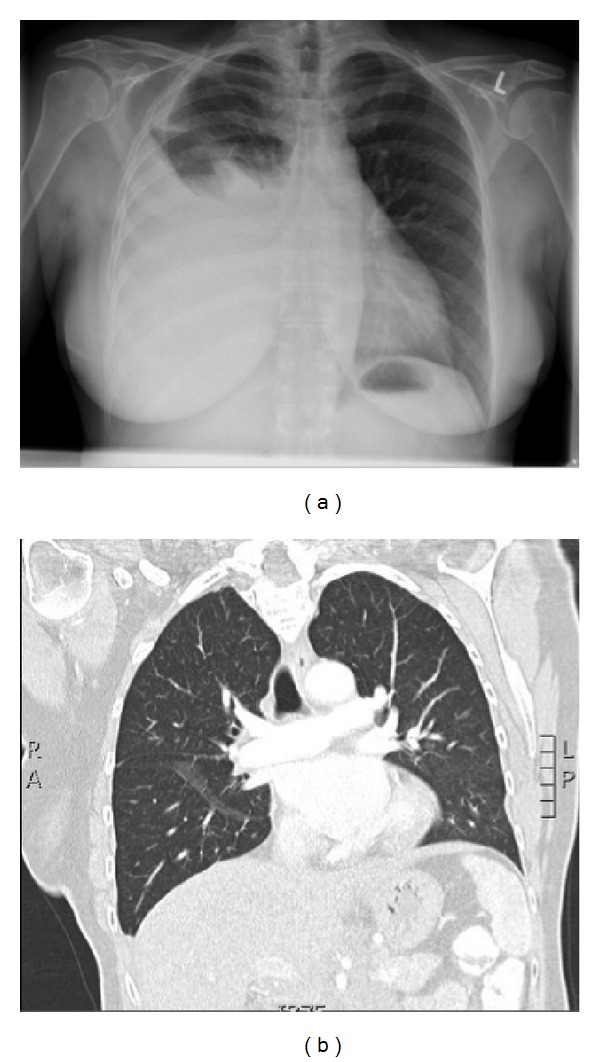
Chest X-ray before radiotherapy: large amount of pleural fluid (right) (a) with complete remission 3 months after radiotherapy on CT thorax (b).

**Figure 2:**
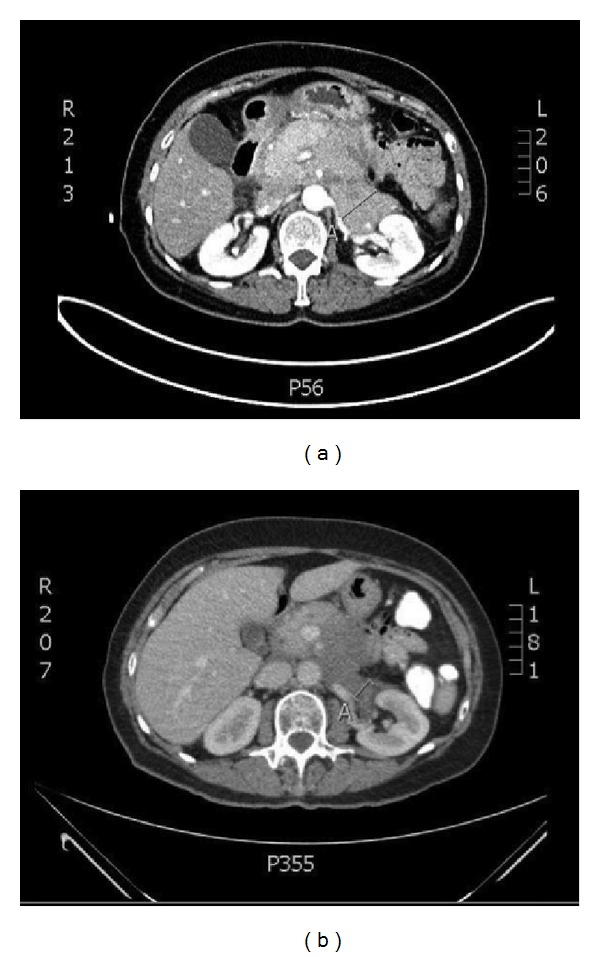
CT abdomen shows a confluent lymphoma mass commencing at the celiac trunk and extending inferiorly to the pelvic outlet (a) before and (b) after radiotherapy.

In August 2013, a 63-year-old woman presented with a gradual onset of decreased exercise tolerance, dyspnea, and a heavy feeling in the chest. Otherwise she was in good health with no relevant concomitant diseases. She denied fever, hemoptysis, weight loss, or other constitutional symptoms. There was no drug, alcohol, or nicotine abuse. She took no medication and there was no recent surgery or trauma. On physical examination we found a decreased breath sound over the right hemithorax and dullness to percussion at the right side of the thorax. There were no signs of peripheral oedema or ascites. She did not have any peripheral lymphadenopathy or enlarged spleen and liver. Laboratory values were normal for a white blood cell count of 5.9 × 10^9^/L, a hematocrit of 44%, and a platelet count of 192 × 10^9^/L. Her creatinine was within normal range and albumin was 42 g/L, with a total protein of 76 g/dL. Furthermore lactate dehydrogenase and tests for glutamic-oxaloacetic transaminase, serum glutamic-pyruvic transaminase, alkaline phosphatase, and bilirubin were all normal. The posteroanterior chest radiograph on admission confirmed a large right pleural effusion (Figure 1). On admission, the effusion was drained showing a turbid and milky white appearance. Biochemical tests on the fluid showed a high triglyceride content (79 mmol/L) in the form of chylomicrons with cholesterol levels as low as 4.6 mmol/L. Further cytological analysis showed the presence of lymphocytosis but no malignant cells. Flow cytometry confirmed the absence of B-cell or T-cell lymphoma. Further imaging with PET-CT was initiated searching for the underlying disease. This revealed a PET positive bulky intraperitoneal tumour around the abdominal aorta and pancreas extending to the pelvic inlet (Figure 2). There was a heterogeneous contrast enhancement and clear encasement of nearby arteries and veins. No enlarged mediastinal lymph nodes or suspect gynaecological malignancy was seen. In our case, the disruption occurred apparently below the fifth thoracic vertebra, and with the thoracic duct being on the right side of the vertebral column a right-sided chylothorax developed. The differential diagnosis of lymphoma or pancreatic carcinoma was made. Biopsy from the large epigastric mass revealed a mixed lymphoid cell population with a significant amount of CD 20 positive B-cells next to a small population of CD 3 and CD 5 positive T-cells. CD 23 staining was negative and only very few neutrophilic granulocytes showed CD 15 positivity. In the CD 30 staining there was no increase in blast cells. Fluorescent* in situ* hybridization detected IGH/bcl2 translocation and the final diagnosis follicular lymphoma grade 1-2 was made. Consequently a bone marrow biopsy was planned showing no arguments for lymphoma localization or stage IV disease. In conclusion she was diagnosed with a stage IIE retroperitoneal follicular lymphoma. Despite the high tumor burden, only her age (>60 years) was an adverse prognostic factor according to the FLIPI index. The patient is considered low risk with an overall survival at 10 years that is estimated to be 70%. Although the FLIPI is a validated prognostic index for follicular lymphoma, there is no consensus on any of the treatment approaches. In an initial attempt to reduce chyle production the patient received an oral diet with medium-chain triglycerides (MCT) [21, 22]. Unfortunately, the pleural effusion and its associated respiratory symptoms reoccurred. After discussion at the multidisciplinary board we decided to treat her with low dose radiotherapy. We argued that her low FLIPI indicates that the optimal treatment has to avoid toxicity and preserve quality of life.

**Figure 3:**
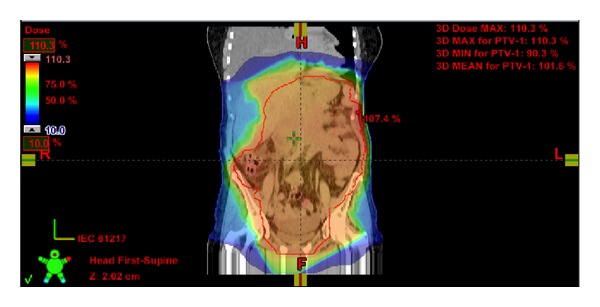
3D dose distribution for radiotherapy planning of abdominal lymphoma.

After CT-based simulation she was treated to a total dose of 4 Gy given in 2 fractions of 2 Gy per fraction over 2 consecutive days. Radiation was delivered with a linear accelerator with 6 MV photons and anterior-posterior/posterior-anterior (AP/PA) fields (Figure 3). The low irradiation dose was chosen because of the large treatment volume and no clear known dose-response relationship to obtain a good long-term control of symptoms [23, 24]. We decided to limit the treatment volume to the abdomen. We mention one case report of a patient with lymphoma-associated chylothorax where chemotherapy resulted in good response for mediastinal lymphadenopathy but progressive disease in the abdomen. Noteworthy, also the pleural effusion remained present [16]. As expected, treatment was tolerated excellently with a clear clinical response four weeks after radiotherapy. Until present, she has no respiratory problems and even rides her bicycle for 40 kilometers without any problems. Two months after treatment restaging CT showed a partial remission of abdominal lymph nodes and no presence of chyle fluid in the thorax. Since she remained asymptomatic we decided to defer further systemic treatment. Of note, no recurrence of pleural effusion occurred during the complete 6 months of follow-up after radiotherapy.

## 3. Discussion

In summary, chylothorax is a rare condition with no strict guidelines concerning therapy [8, 12, 25–28]. We present, to our knowledge, the first case report of a 63-year-old patient presenting with right-sided chylothorax caused by a concomitantly diagnosed follicular lymphoma and successfully treated with low dose radiotherapy (2 × 2 Gy). About one month after treatment the patient already experienced a good clinical response with complete radiographic remission of the pleural effusion after two months. During follow-up no recurrence of symptoms or progression of disease was noted with consequently no need to start systemic treatment.

A large number of novel agents are in the clinical pipeline for treatment of follicular lymphoma but radiotherapy remains a good remedy to extend the duration of remission without adding any further burden of toxicity [18, 20, 29–31]. Follicular lymphoma has long been recognized as more radiosensitive than other lymphoma subtypes [32]. Although a higher dose of radiotherapy (24 Gy) seems to have a superior progression-free survival, no difference in overall survival was noted [32]. In our patient we expected a higher incidence of grade 3 or higher gastrointestinal toxicity with the high dose radiotherapy and opted for a pragmatic alternative of 4 Gy. Localized low dose radiotherapy appears to induce apoptosis and this follicular lymphoma cell death may then elicit a host immune response mediated by macrophages and dendritic cells. This exquisite radiation-induced apoptosis and subsequent immune response may underlie the durability of remission of chylothorax in patients. Other treatment options are rituximab with or without chemotherapy (CHOP or CVP regimens) but these are associated with a higher toxicity rate. In future this low dose radiotherapy in combination with other drugs such as rituximab might result in both optimum local control and carrying the advantage of systemic effects for more widespread disease control [32]. It is becoming clear that the therapy for follicular lymphoma needs to be adapted to the patient's individual status [20].

To date, this unique manifestation of lymphoma-associated disease does not always warrant an aggressive treatment to resolve clinical symptoms and radiotherapy may play a vital role [1, 33, 34]. We advise an interdisciplinary case management of lymphoma-associated chylothorax, including hematologists, radiation oncologist, and thoracic surgeons. To conclude, radiotherapy to the abdominal lymphoma location has proven to be an effective nontoxic treatment to induce a complete remission of chylothorax [35].

## References

[1] Janjetovic S, Janning M, Daukeva L, Bokemeyer C, Fiedler W (2013). Chylothorax in a patient with hodgkin's lymphoma: a case report and review of the literature. *Tumori*.

[2] Scholz GA, Sirbu H, Semrau S, Anders K, MacKensen A, Spriewald BM (2011). Persisting right-sided chylothorax in a patient with chronic lymphocytic leukemia: a case report. *Journal of Medical Case Reports*.

[3] Thomas LC, Maida MJ, Martinez-Outschoorn U, Filicko-O’Hara J, Morris GJ (2011). Chronic lymphocytic leukemia/small lymphocytic lymphoma with pancytopenia and chylothorax. *Seminars in Oncology*.

[4] Menon BS, Juraida E, Mahfuzah M, Hishamshah I (2006). Bilateral chylothorax in paediatric acute lymphoblastic leukaemia. *British Journal of Haematology*.

[5] Gabrys K, Kacprzak G (1999). A case of massive chylothorax during the course of malignant lymphoma. *Polskie Archiwum Medycyny Wewnętrznej*.

[6] Asuquo BJ, Gould GA (2004). Recurrent chylothorax in a patient with non-Hodgkins lymphoma: case report. *East African Medical Journal*.

[7] Nonami A, Yokoyama T, Takeshita M, Ohshima K, Kubota A, Okamura S (2004). Human herpes virus 8-negative primary effusion lymphoma (PEL) in a patient after repeated chylous ascites and chylothorax. *Internal Medicine*.

[8] Ohyama S, Bando K, Hasegawa Y, Taniguti M, Shirayma H (1998). Chylothorax resulting from malignant non Hodgkin’s lymphoma. *Nihon Kokyuki Gakkai Zasshi*.

[9] Naseer A, Saeed W (2003). Chylothorax in a case of Non-Hodgkin’s lymphoma. *Journal of the College of Physicians and Surgeons Pakistan*.

[10] Iqbal MH, Smith PR, Bande S (2009). Chylothorax due to angioimmunoblastic T-cell lymphoma. *Internal Medicine Journal*.

[11] Doerr CH, Allen MS, Nichols FC, Ryu JH (2005). Etiology of chylothorax in 203 patients. *Mayo Clinic Proceedings*.

[12] O'Callaghan AM, Mead GM (1995). Chylothorax in lymphoma: mechanisms and management. *Annals of Oncology*.

[13] Sassoon CS, Light RW (1985). Chylothorax and pseudochylothorax. *Clinics in Chest Medicine*.

[14] Etonyeaku AC, Akinsanya OO, Ariyibi O, Aiyeyemi AJ (2012). Chylothorax from bilateral primary burkitt's lymphoma of the ovaries: a case report. *Case Reports in Obstetrics and Gynecology*.

[15] Khosravi A, Anjidani AA (2009). Spontaneous recovery of chylothorax caused by lymphoma. *Hematology Oncology and Stem Cell Therapy*.

[16] Takami A, Fujimura M, Nakao S (1993). A case of chylothorax resulting from malignant lymphoma—pathogenesis of chylothorax a new concept. *Japanese Journal of Thoracic Diseases*.

[17] Solal-Céligny P, Roy P, Colombat P (2004). Follicular lymphoma international prognostic index. *Blood*.

[18] Michallet AS, Lebras LL, Bauwens DD (2013). Early stage follicular lymphoma: what is the clinical impact of the first-line treatment strategy?. *Journal of Hematology and Oncology*.

[19] Relander T, Johnson NA, Farinha P, Connors JM, Sehn LH, Gascoyne RD (2010). Prognostic factors in follicular lymphoma. *Journal of Clinical Oncology*.

[20] Tageja N, Padheye S, Dandawate P, Al-Katib A, Mohammad RM (2009). New targets for the treatment of follicular lymphoma. *Journal of Hematology and Oncology*.

[21] Martínez Brocca MA, García García-Doncel L, Pereira Cunill JL, Ortegón Alcaide S, Martino Galiano ML, García Luna PP (2002). Nutritional support in chylothorax secondary to lymphoma. *Nutricion Hospitalaria*.

[22] Jensen GL, Mascioli EA, Meyer LP (1989). Dietary modification of chyle composition in chylothorax. *Gastroenterology*.

[23] Haas RL, Poortmans P, de Jong D (2003). High response rates and lasting remissions after low-dose involved field radiotherapy in indolent lymphomas. *Journal of Clinical Oncology*.

[24] Ganem G, Cartron G, Girinsky T, Haas RLM, Cosset JM, Solal-Celigny P (2010). Localized low-dose radiotherapy for follicular lymphoma: history, clinical results, mechanisms of action, and future outlooks. *International Journal of Radiation Oncology Biology Physics*.

[25] McGrath EE, Blades Z, Anderson PB (2010). Chylothorax: Aetiology, diagnosis and therapeutic options. *Respiratory Medicine*.

[26] Evans J, Clark MF, Mincher L, Varney VA (2003). Chylous effusions complicating lymphoma: a serious event with octreotide as a treatment option. *Hematological Oncology*.

[27] Marts BC, Naunheim KS, Fiore AC (1992). Conservative versus surgical management of chylothorax. *The American Journal of Surgery*.

[28] Fahimi H, Casselman FP, Mariani MA, Van Boven WJ, Knaepen PJ, van Swieten HA (2001). Current management of postoperative chylothorax. *Annals of Thoracic Surgery*.

[29] Wilder RB, Jones D, Tucker SL (2001). Long-term results with radiotherapy for stage i-ii follicular lymphomas. *International Journal of Radiation Oncology Biology Physics*.

[30] Friedberg JW, Byrtek M, Link BK (2012). Effectiveness of first-line management strategies for stage i follicular lymphoma: analysis of the national lymphocare study. *Journal of Clinical Oncology*.

[31] Montoto S (2012). Management of localized-stage follicular lymphoma: changing the paradigm?. *Journal of Clinical Oncology*.

[32] Hoskin PJ, Kirkwood AA, Popova B (2014). 4 gy versus 24 gy radiotherapy for patients with indolent lymphoma (fort): A randomised phase 3 non-inferiority trial. *The Lancet Oncology*.

[33] Barosi G, Carella A, Lazzarino M (2005). Management of nodal indolent (non marginal-zone) non-hodgkin's lymphomas: practice guidelines from the Italian society of hematology, Italian society of experimental hematology and italian group for bone marrow transplantation. *Haematologica*.

[34] Pugh TJ, Ballonoff A, Newman F, Rabinovitch R (2010). Improved survival in patients with early stage low-grade follicular lymphoma treated with radiation: a surveillance, epidemiology, and end results database analysis. *Cancer*.

[35] Gerstein J, Kofahl-Krause D, Frühauf J, Bremer M (2008). Complete remission of a lymphoma-associated chylothorax by radiotherapy of the celiac trunk and thoracic duct. *Strahlentherapie und Onkologie*.

